# Annotations

**Published:** 1895-01-05

**Authors:** 


### Annotations.

### Cholera Inoculation: More Evidence.

Dr. Simpson, of Calcutta, has furnished a contribution to the *Practitioner* of considerable importance. Additional experiments have been made during the year in the districts of Cawnpore, Gya, Dinapore, Lucknow, and Calcutta. The more important of these may be briefly summarised. Cholera attacked the Munster Regiment at Cawnpore during the severe epidemic of the present year. Of the 797 non-inoculated soldiers 19 took cholera, and 15 died. Of the 75 inoculated none were attacked. A still more important series of experiments was performed on the prisoners at the Gya jail. During the epidemic there were present in jail about 207 inoculated prisoners, as against 202 not inoculated. Among the inoculated there were 8 cases, with 5 deaths, whilst among the non-inoculated there were 20 cases, with 10 deaths. Dr. Simpson refers to the apparent failure of the inoculations in the Lancashire regiment at Lucknow, which we reported in these pages. He states, however, that even there the failure was less complete than has been announced, the percentage of deaths among the non-inoculated being 12·34, as against 9·77 for the inoculated. We cannot but feel that many more experiments are required before any generalisations of permanent value can possibly be made.

### The Domestic Filter.

The breaking of images and the uprooting of old faiths has often been looked upon as one of the first and most necessary steps in reformation, and from that point of view Dr. Sims Woodhead and Dr. Cartwright Wood must be regarded as the very advance guard of sanitary reform. At the request of the *British Medical Journal* they have been investigating our domestic filters, household gods to which we have long turned for protection against cholera and other epidemics, and as the result of their researches they have become iconoclasts pure and simple. Our images are broken, and we are left surrounded by the fragments of our discarded filters, convicted of having drunk, for all these years, innumerable micro-organisms, and, in our deep respect for sanitary science, filled with doubt and wonder that we are yet alive. Nevertheless the facts reported are very striking, and deserve the most careful consideration on the part both of householders and hospital authorities. What was found in almost every case was that, although during the first day or two, when tested with ordinary tap water, the number of micro-organisms was considerably reduced, by the third or fourth day they grew through the filtering material, and appeared in the filtered water in far larger numbers than in that which had not been touched. It is obvious that the filtering medium which is intended to clear out all impurities, and which does very effectually remove all visible opacity, really acts as a breeding ground for the invisible forms of life, so that all the dangerous impurities are actually increased by the process. That this happened in regard to certain forms of block filter has long been known, and has been made much of by manufacturers of other kinds, who in the pride of ignorance have advertised that they were not as other filter makers, and that they changed their filtering medium every three months, whereas now comes out the unwelcome truth that they are as great sinners as the rest, and that when their filters have been used for a few days, water which on going in contained only a modest forty or fifty organisms per cubic centimetre, comes out with four or five hundred, or, as was generally the case, with micro-organisms innumerable. Only three filters were found which remained active after a few days of use, and they were all constructed on the same plan, viz., tubes of porcelain or very slightly porous material through which the water was made to pass. The three good filters then, according to this report, are the "Filtre Chamberland," the "Berkefeld Filter," and the "Aeri-Filtre-Mallie." These all seem to stand the test, but unfortunately we are immediately met with another difficulty in the fact that they filter very slowly at the low pressure which alone is possible in the domestic apparatus, and the final word is that, so far as the inquiry has yet gone, the safest kind of filter, in fact the only kind which combines safety with utility, is one or other of these tube filters attached to a tap, so as to operate under a certain pressure, and thus provide a more copious supply than would be possible in the low-pressure form. The result is sadly disappointing, and it must cause much vexation to many careful housekeepers to be told that all the endless trouble they have taken for so many years in the cleaning of their filters has been so much labour thrown away.

### "Men's Children."

What can be the meaning of the expression "men's children"? The careful reader of his *Times* can answer the question. It is a woman's expression; and according to a correspondent in the leading journal, who signs herself "A Woman Worker," there is growing up a class of young women among us who commonly speak of children as "Men's Children," and who think it is degrading to be bearing and bringing up children when they might be doing "higher work." Now this is both amusing and ridiculous. One of the correspondents, who deals with the topic of "Our Daughters" in the *Times*, expresses the conviction that their business is to marry; but that their ambition to compete with men in men's fields of work so cuts down male incomes that marriage cannot be afforded. This seems to us to be at least one of the places where the shoe pinches. Whatever be the teaching of those who essay the guidance of the "New Woman," there is no doubt that marriage and child-bearing will always remain the true destiny of women. The only effect produced by the non-marrying faddist is that of securing her own futility and extinction. That she should ever change the constitution of the human world is an absurd impossibility. Women, as well as men, will do well to accept the inevitable with a good grace.

